# Supplementary material for: Heparin-based hydrogel scaffolding alters the transcriptomic profile and increases the chemoresistance of MDA-MB-231 triple-negative breast cancer cells
Source: Biomater Sci. 2020 Feb 13;8(10):2786–96. doi: 10.1039/c9bm01481k (PMC7497406; doi:10.1039/c9bm01481k)
Supplement: Supplementary file 2 [file BM-008-C9BM01481K-s002.zip › Supplementary File 4/HP-BhydrogelvControl/Pathways/my_analysis.Gsea.1545201079907/HALLMARK_INFLAMMATORY_RESPONSE.html]

Details for gene set HALLMARK\_INFLAMMATORY\_RESPONSE[GSEA]

|  || Dataset | expr.class.cls#HYD\_versus\_CONTROL.class.cls#HYD\_versus\_CONTROL\_repos |
| Phenotype | class.cls#HYD\_versus\_CONTROL\_repos |
| Upregulated in class | CONTROL |
| GeneSet | HALLMARK\_INFLAMMATORY\_RESPONSE |
| Enrichment Score (ES) | -0.15886591 |
| Normalized Enrichment Score (NES) | -0.7270163 |
| Nominal p-value | 1.0 |
| FDR q-value | 0.9660864 |
| FWER p-Value | 1.0 |
Table: GSEA Results Summary

  

Fig 1: Enrichment plot: HALLMARK\_INFLAMMATORY\_RESPONSE      
 Profile of the Running ES Score & Positions of GeneSet Members on the Rank Ordered List

  

| PROBE | DESCRIPTION (from dataset) | GENE SYMBOL | GENE\_TITLE | RANK IN GENE LIST | RANK METRIC SCORE | RUNNING ES | CORE ENRICHMENT || 1 | SCARF1 | na |  |  | 303 | 2.668 | 0.0210 | No |
| 2 | GABBR1 | na |  |  | 1078 | 1.817 | 0.0055 | No |
| 3 | GPR132 | na |  |  | 1115 | 1.789 | 0.0283 | No |
| 4 | CHST2 | na |  |  | 1302 | 1.689 | 0.0419 | No |
| 5 | PVR | na |  |  | 1551 | 1.551 | 0.0503 | No |
| 6 | PTGER4 | na |  |  | 1903 | 1.406 | 0.0514 | No |
| 7 | PCDH7 | na |  |  | 2210 | 1.271 | 0.0529 | No |
| 8 | TNFSF9 | na |  |  | 2848 | 1.072 | 0.0343 | No |
| 9 | SLC31A2 | na |  |  | 2970 | 1.044 | 0.0424 | No |
| 10 | IRF7 | na |  |  | 3003 | 1.036 | 0.0550 | No |
| 11 | RAF1 | na |  |  | 3157 | 0.999 | 0.0608 | No |
| 12 | SPHK1 | na |  |  | 3452 | 0.927 | 0.0582 | No |
| 13 | NFKB1 | na |  |  | 3668 | 0.878 | 0.0590 | No |
| 14 | MYC | na |  |  | 3771 | 0.854 | 0.0655 | No |
| 15 | RASGRP1 | na |  |  | 3920 | 0.828 | 0.0692 | No |
| 16 | GP1BA | na |  |  | 4183 | 0.773 | 0.0661 | No |
| 17 | ICOSLG | na |  |  | 4783 | 0.662 | 0.0439 | No |
| 18 | TPBG | na |  |  | 4814 | 0.656 | 0.0513 | No |
| 19 | EDN1 | na |  |  | 4838 | 0.651 | 0.0591 | No |
| 20 | FZD5 | na |  |  | 4966 | 0.633 | 0.0612 | No |
| 21 | CCR7 | na |  |  | 5027 | 0.622 | 0.0667 | No |
| 22 | LIF | na |  |  | 5094 | 0.614 | 0.0717 | No |
| 23 | LDLR | na |  |  | 5135 | 0.608 | 0.0780 | No |
| 24 | SEMA4D | na |  |  | 5207 | 0.598 | 0.0825 | No |
| 25 | RGS16 | na |  |  | 5502 | 0.552 | 0.0747 | No |
| 26 | HBEGF | na |  |  | 5567 | 0.542 | 0.0789 | No |
| 27 | IRF1 | na |  |  | 5648 | 0.533 | 0.0820 | No |
| 28 | TNFRSF1B | na |  |  | 5672 | 0.529 | 0.0881 | No |
| 29 | ADRM1 | na |  |  | 5890 | 0.501 | 0.0837 | No |
| 30 | GCH1 | na |  |  | 6157 | 0.463 | 0.0761 | No |
| 31 | ATP2A2 | na |  |  | 6241 | 0.453 | 0.0780 | No |
| 32 | SLC4A4 | na |  |  | 6588 | 0.405 | 0.0655 | No |
| 33 | CSF3 | na |  |  | 6738 | 0.388 | 0.0630 | No |
| 34 | NLRP3 | na |  |  | 6756 | 0.386 | 0.0675 | No |
| 35 | CXCL10 | na |  |  | 6890 | 0.372 | 0.0656 | No |
| 36 | IL18 | na |  |  | 7027 | 0.356 | 0.0634 | No |
| 37 | SLC7A1 | na |  |  | 7059 | 0.353 | 0.0667 | No |
| 38 | IL6 | na |  |  | 7244 | 0.333 | 0.0616 | No |
| 39 | BTG2 | na |  |  | 7702 | 0.284 | 0.0416 | No |
| 40 | CX3CL1 | na |  |  | 8580 | 0.196 | -0.0017 | No |
| 41 | ADORA2B | na |  |  | 8611 | 0.191 | -0.0006 | No |
| 42 | ACVR2A | na |  |  | 8850 | 0.159 | -0.0109 | No |
| 43 | F3 | na |  |  | 9090 | 0.142 | -0.0215 | No |
| 44 | SCN1B | na |  |  | 9124 | 0.137 | -0.0213 | No |
| 45 | IL1B | na |  |  | 9136 | 0.136 | -0.0200 | No |
| 46 | IL4R | na |  |  | 9138 | 0.135 | -0.0182 | No |
| 47 | CYBB | na |  |  | 9295 | 0.118 | -0.0248 | No |
| 48 | LAMP3 | na |  |  | 9393 | 0.105 | -0.0284 | No |
| 49 | CMKLR1 | na |  |  | 9419 | 0.102 | -0.0283 | No |
| 50 | IRAK2 | na |  |  | 9448 | 0.097 | -0.0284 | No |
| 51 | ADM | na |  |  | 9646 | 0.081 | -0.0377 | No |
| 52 | IL1A | na |  |  | 9651 | 0.080 | -0.0368 | No |
| 53 | PTPRE | na |  |  | 9844 | 0.059 | -0.0460 | No |
| 54 | CXCR6 | na |  |  | 9862 | 0.057 | -0.0461 | No |
| 55 | IL15RA | na |  |  | 9909 | 0.053 | -0.0478 | No |
| 56 | ICAM1 | na |  |  | 9949 | 0.046 | -0.0492 | No |
| 57 | ABCA1 | na |  |  | 10121 | 0.026 | -0.0578 | No |
| 58 | EBI3 | na |  |  | 10141 | 0.023 | -0.0585 | No |
| 59 | BEST1 | na |  |  | 10393 | 0.007 | -0.0716 | No |
| 60 | SLC11A2 | na |  |  | 10509 | -0.006 | -0.0775 | No |
| 61 | P2RX4 | na |  |  | 10928 | -0.055 | -0.0987 | No |
| 62 | GNA15 | na |  |  | 10989 | -0.064 | -0.1009 | No |
| 63 | LY6E | na |  |  | 11120 | -0.081 | -0.1067 | No |
| 64 | PLAUR | na |  |  | 11509 | -0.112 | -0.1254 | No |
| 65 | CD14 | na |  |  | 11546 | -0.118 | -0.1257 | No |
| 66 | TAPBP | na |  |  | 11603 | -0.126 | -0.1269 | No |
| 67 | CD82 | na |  |  | 11683 | -0.136 | -0.1292 | No |
| 68 | CDKN1A | na |  |  | 11746 | -0.145 | -0.1304 | No |
| 69 | RNF144B | na |  |  | 11771 | -0.149 | -0.1296 | No |
| 70 | SLC31A1 | na |  |  | 11916 | -0.165 | -0.1349 | No |
| 71 | LPAR1 | na |  |  | 11939 | -0.168 | -0.1337 | No |
| 72 | KIF1B | na |  |  | 12121 | -0.189 | -0.1406 | No |
| 73 | TNFRSF9 | na |  |  | 12175 | -0.197 | -0.1406 | No |
| 74 | FPR1 | na |  |  | 12252 | -0.207 | -0.1418 | No |
| 75 | NFKBIA | na |  |  | 12331 | -0.218 | -0.1429 | No |
| 76 | CCL2 | na |  |  | 12375 | -0.222 | -0.1420 | No |
| 77 | ITGB8 | na |  |  | 12579 | -0.232 | -0.1495 | No |
| 78 | AHR | na |  |  | 12759 | -0.253 | -0.1554 | Yes |
| 79 | CCL20 | na |  |  | 12777 | -0.255 | -0.1527 | Yes |
| 80 | TLR1 | na |  |  | 12783 | -0.255 | -0.1495 | Yes |
| 81 | TLR3 | na |  |  | 12803 | -0.258 | -0.1469 | Yes |
| 82 | TLR2 | na |  |  | 12836 | -0.262 | -0.1449 | Yes |
| 83 | KLF6 | na |  |  | 12855 | -0.265 | -0.1422 | Yes |
| 84 | ACVR1B | na |  |  | 13007 | -0.284 | -0.1462 | Yes |
| 85 | IL1R1 | na |  |  | 13023 | -0.288 | -0.1430 | Yes |
| 86 | RHOG | na |  |  | 13032 | -0.288 | -0.1395 | Yes |
| 87 | CXCL11 | na |  |  | 13037 | -0.289 | -0.1357 | Yes |
| 88 | RELA | na |  |  | 13082 | -0.296 | -0.1339 | Yes |
| 89 | ITGA5 | na |  |  | 13121 | -0.302 | -0.1317 | Yes |
| 90 | CD40 | na |  |  | 13309 | -0.327 | -0.1370 | Yes |
| 91 | MXD1 | na |  |  | 13509 | -0.354 | -0.1426 | Yes |
| 92 | INHBA | na |  |  | 13523 | -0.357 | -0.1383 | Yes |
| 93 | P2RY2 | na |  |  | 13598 | -0.368 | -0.1371 | Yes |
| 94 | SLC7A2 | na |  |  | 13664 | -0.379 | -0.1353 | Yes |
| 95 | LYN | na |  |  | 13668 | -0.380 | -0.1302 | Yes |
| 96 | ATP2B1 | na |  |  | 14037 | -0.422 | -0.1437 | Yes |
| 97 | IFITM1 | na |  |  | 14071 | -0.427 | -0.1395 | Yes |
| 98 | NMI | na |  |  | 14136 | -0.432 | -0.1369 | Yes |
| 99 | OSMR | na |  |  | 14334 | -0.469 | -0.1407 | Yes |
| 100 | PSEN1 | na |  |  | 14410 | -0.478 | -0.1380 | Yes |
| 101 | ABI1 | na |  |  | 14438 | -0.481 | -0.1328 | Yes |
| 102 | PTGER2 | na |  |  | 14456 | -0.483 | -0.1270 | Yes |
| 103 | TIMP1 | na |  |  | 14560 | -0.499 | -0.1255 | Yes |
| 104 | RTP4 | na |  |  | 14854 | -0.544 | -0.1334 | Yes |
| 105 | HRH1 | na |  |  | 14956 | -0.555 | -0.1310 | Yes |
| 106 | IL15 | na |  |  | 15098 | -0.579 | -0.1304 | Yes |
| 107 | SGMS2 | na |  |  | 15107 | -0.581 | -0.1228 | Yes |
| 108 | BST2 | na |  |  | 15210 | -0.597 | -0.1199 | Yes |
| 109 | PDE4B | na |  |  | 15522 | -0.656 | -0.1271 | Yes |
| 110 | IL7R | na |  |  | 15528 | -0.657 | -0.1183 | Yes |
| 111 | TNFSF15 | na |  |  | 15540 | -0.659 | -0.1098 | Yes |
| 112 | IFNGR2 | na |  |  | 15618 | -0.672 | -0.1046 | Yes |
| 113 | EMP3 | na |  |  | 15769 | -0.696 | -0.1028 | Yes |
| 114 | ATP2C1 | na |  |  | 16035 | -0.755 | -0.1063 | Yes |
| 115 | EIF2AK2 | na |  |  | 16137 | -0.775 | -0.1009 | Yes |
| 116 | IFNAR1 | na |  |  | 16431 | -0.839 | -0.1046 | Yes |
| 117 | ITGB3 | na |  |  | 16433 | -0.839 | -0.0931 | Yes |
| 118 | TNFSF10 | na |  |  | 16587 | -0.869 | -0.0891 | Yes |
| 119 | RIPK2 | na |  |  | 16616 | -0.878 | -0.0784 | Yes |
| 120 | HAS2 | na |  |  | 16806 | -0.929 | -0.0755 | Yes |
| 121 | EREG | na |  |  | 17124 | -1.024 | -0.0780 | Yes |
| 122 | NDP | na |  |  | 17477 | -1.143 | -0.0807 | Yes |
| 123 | DCBLD2 | na |  |  | 17598 | -1.196 | -0.0704 | Yes |
| 124 | GNAI3 | na |  |  | 17910 | -1.337 | -0.0683 | Yes |
| 125 | SRI | na |  |  | 17999 | -1.383 | -0.0538 | Yes |
| 126 | MET | na |  |  | 18018 | -1.394 | -0.0355 | Yes |
| 127 | NAMPT | na |  |  | 18342 | -1.596 | -0.0304 | Yes |
| 128 | HIF1A | na |  |  | 18348 | -1.603 | -0.0085 | Yes |
| 129 | CD55 | na |  |  | 18406 | -1.643 | 0.0112 | Yes |
| 130 | CCRL2 | na |  |  | 18912 | -2.189 | 0.0150 | Yes |
Table: GSEA details [plain text format]

  

Fig 2: HALLMARK\_INFLAMMATORY\_RESPONSE      
 Blue-Pink O' Gram in the Space of the Analyzed GeneSet

  

Fig 3: HALLMARK\_INFLAMMATORY\_RESPONSE: Random ES distribution      
 Gene set null distribution of ES for **HALLMARK\_INFLAMMATORY\_RESPONSE**
